# Extracorporeal membrane oxygenation treatment of a H7N9-caused respiratory failure patient with mechanical valves replacement history: A case report

**DOI:** 10.1097/MD.0000000000005052

**Published:** 2016-10-07

**Authors:** Linfeng Qian, Junnan Zheng, Hongfei Xu, Liping Shi, Lanjuan Li

**Affiliations:** a Department of Cardiothoracic Surgery, the First Affiliated Hospital, Zhejiang University; b Collaborative Innovation Center for Diagnosis and Treatment of Infectious Diseases, the First Affiliated Hospital, Zhejiang University, Hangzhou, China.

**Keywords:** acute respiratory distress syndrome, extracorporeal membrane oxygenation, intensive care

## Abstract

**Background:**

Patients with respiratory failure caused by H7N9 may benefit from veno-venous, veno-arterial, and veno-veno-arterial extracorporeal membrane oxygenation (ECMO) support.

**Case summary:**

A 55-year-old male patient was suffering from H7N9-caused acute respiratory distress syndrome (ARDS). He had a mechanical mitral and aortic valve replacement surgery and was using warfarin for anticoagulation. After prolonged mechanical ventilation, oxygen saturation was not improved. Veno-veno ECMO was then applied. After 16 days of extracorporeal life support, the patient successfully weaned from ECMO, with relatively good pulmonary recovery.

**Conclusion:**

This report demonstrates that ECMO support can help treating life-threatening diseases such as H7N9-associated ARDS. Because of his special mitral and aortic valve replacement surgery history and long duration of mechanical ventilation before ECMO, we report it as a separate case, hoping to provide some reference for ECMO treatment.

## 1 Introduction

The influenza A (H7N9) virus has now become the most prevalent avian influenza virus affecting humans in China since its appearance in the spring of 2013.^[1,2]^ Similar to other severe influenza virus infection, H7N9 typically causes severe pneumonia not responding to antibiotics covering typical and atypical respiratory pathogens, and can lead to extrapulmonary complications.^[3]^ Acute respiratory failure is one of the most severe complications, of which approximately one-third have resulted in death. Patients with respiratory failure caused by H7N9 may benefit from veno-venous (v-v), veno-arterial (v-a), and veno-veno-arterial (v-v-a) extracorporeal membrane oxygenation (ECMO) support. Here we report on our initial experience of treating 1 special patient with respiratory failure with v-v ECMO.

## 2 Case summary

**Figure 1:**
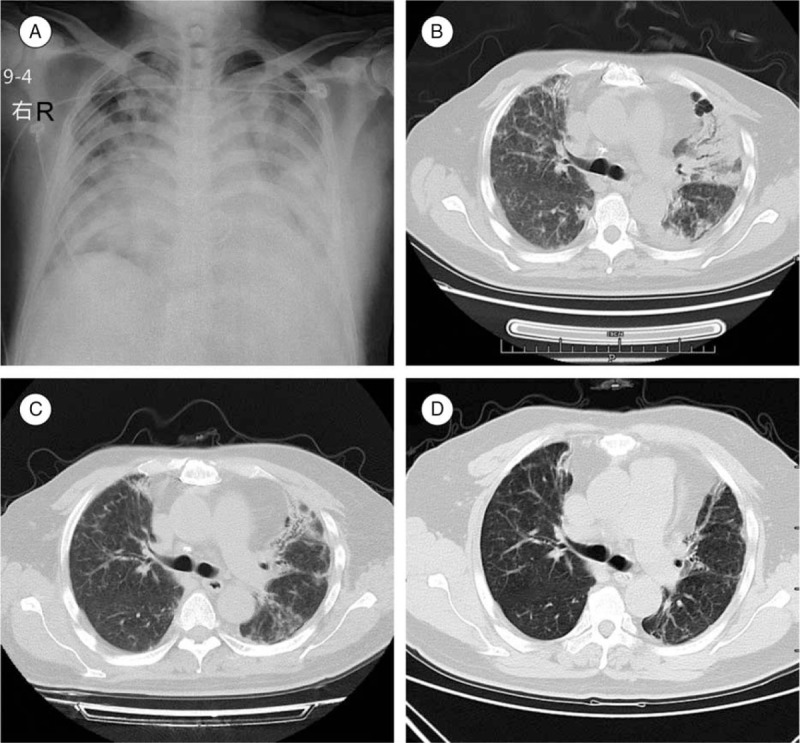
(A) Chest X-ray at admission showed diffuse inflammation and fibrosis of both lungs. (B) CT scan at admission revealed severe lung infection with fibrosis, and the heart in postoperative of mechanical valve replacement changes. (C) CT scan at discharge showed moderate fibrosis and inflammation, improved significantly comparing to CT at admission. (D) 1-year follow-up of chest CT showed multiple chronic infections with fibrosis of both lungs. CT = computed tomography.

A 55-year-old male was admitted to our hospital due to fever for 8 days and cough for 6 days on January 16, 2014. He denied history of exposure to poultry. His medical history showed that 5 years ago he underwent a mechanical mitral and aortic valve replacement surgery due to rheumatic heart valve disease, thereafter he took warfarin (orion corporation) 3 mg qd to maintain international normalized ratio in 2.5 to 3.0. On physical examination, a few moist rales have been audible over both lung bases. After admission, he showed continued fever with no therapeutic effect to broad spectrum antibiotic, and chest X-ray along with computed tomography (CT) scan revealed progress of lung infection (Fig. 1A and B). The troponin-I result was negative; however, the CK-MB level was 0.78 μg/L. Electrocardiogram demonstrated first degree atrioventricular block. Thus the primary diagnosis of pulmonary infection with probable myocarditis was made. Two days after his hospitalization, H7N9 influenza virus RNA was detected in this patient and his influenza virus subtype assays was positive for influenza A. At the same day, he began to manifest irritability, difficulty of breathing with his oxygen saturation dropped to 77% to 85% when on mask oxygen-inspiration. Then the endotracheal intubation was executed. Nine days later, his H7N9 influenza virus RNA assay revealed negative. The next day, his oxygen saturation further decreased, the parameter of mechanical ventilation (MV) was fraction of inspiration O_2_ (FiO_2_) 100%, positive end expiratory pressure (PEEP) 17 cm H_2_O, PC mode with 40 cm H_2_O, SPO_2_ 46%. After multidisciplinary discussion, we decided to support the patient with v-v ECMO (V-V ECMO). Under the guide of ultrasound, a 24 # and 20 # catheters (EDWARDS) were successfully cannulated into his right femoral and jugular vein, respectively. Ventilator parameters were FiO_2_ 50%, PEEP 10 cm H_2_O, PC 36 cm H_2_O, tidal volume (Vt) 200 mL, and respiratory rate 20 per min. ECMO parameters were 100% oxygen with flow of 8 L/min, rotational speed 2806 times/min, blood flow 4.26 L/min, and SPO_2_ 94%. Two days after that, we performed tracheotomy for this patient to avoid oral complications without significant bleeding. Gradually, his pulmonary function recovered, and we removed ECMO and MV sequentially. He was discharged from hospital on March 25, 2014. CT scan (Fig. 1C) at discharge showed moderate fibrosis and inflammation, improved significantly comparing to CT at admission. Duration of ECMO support, MV, and hospital stay was 16, 45, and 60 days, respectively.

## 3 Discussion

This patient suffered from a novel viral infection-H7N9 influenza virus. He presented with typical symptoms, cough and fever, for over a week. He also developed pneumonia that did not respond to broad spectrum antibiotics covering for both typical and atypical bacteria. X-ray and CT scan showed that the pneumonia extended to involve both lungs. These typical clinical features along with the laboratory result of influenza virus RNA detection confirmed the definite diagnosis of H7N9 infection. Among various complications of H7N9 infection, acute respiratory distress syndrome (ARDS) most commonly occurred. Other complications included disseminated intravascular coagulation, shock, heart failure, acute kidney injury, rhabdomyolysis, and encephalopathy.^[3]^

This is a big challenge to do ECMO for such a special ARDS patient, because of his history of heart valve replacement with anticoagulation status as well as MV for up to 10 days. Finally the patient was rescued successfully, and we summarize and analyze our experience as follows.

ECMO is a method of life support to maintain cardiopulmonary function. Poor outcomes and a highly unfavorable risk–benefit ratio were seen in early studies, it regained attention in recent years, pivotal progress has been made in the construction of ECMO circuits, rendering them to be more biocompatible, to perform better, and to last longer. Good outcomes of patients who received ECMO as rescue therapy during the previous H1N1 influenza pandemic, in which the latest generation of ECMO was used, reignited interest in ECMO for severe ARDS.^[4,5]^ In the winters of 2009/2010 and 2010/2011, ECMO was used with success in the management of some patients with severe respiratory failure associated with pandemic influenza A (H1N1/09) infection.

ECMO has been proposed as an alternative bridging strategy to MV, and it is employed in 3 types of modality, v-a, v-v, or v-v-a ECMO.^[6]^ Among the 3 types, v-a ECMO is currently used as a bridge to heart transplantation, ventricular assist devices, or potential recovery, for patients with severe cardiac failure.^[7–10]^ Whereas v-v ECMO is employed primarily in patients with severe respiratory failure, in addition to, or as an alternative to MV, bridging to recovery, or lung transplantation.^[11,12]^ However, neither of the 2 types of ECMO support is sufficient for patients of respiratory failure combined with subsequent cardiac failure or upper-body hypoxia. The third type of ECMO, v-v-a ECMO, is defined by having both venous and arterial reinfusion cannulas. It is a rescue procedure for patients already on ECMO who developed cardiac dysfunction on V-V support or worsening hypoxemia on VA support. Our patient had a history of heart surgery, and his echocardiographic assessment before ECMO showed good cardiac function, then the severe respiratory failure should be the primary problem to be solved. Therefore, we preferred the V-V ECMO for treatment and during the 16-day operation time, the patient did not show manifestation of heart failure or abnormal cardiac ultrasound result.

Here we focus on the discussion of V-V ECMO application in ARDS.

So far, there is no unified official guide for the indications and contraindications for ECMO treatment of ARDS. Currently, different centers and experts hold different opinions, especially on the aspect of its contraindications. More consistent indication is PaO_2_/FiO_2_ ratio below 50 mm Hg when FiO_2_ > 80%, while other parameters such as oxygenation index, PH, plateau pressure (P-plat), Vt, and PaCO_2_ have different standards in different regions and time periods.^[13–16]^

There is no completely consistent contraindication. The past 5 years have witnessed a relatively consistent contraindication on ECMO treatment for ARDS stating that MV cannot exceed 7 days.

Our patient suffered sudden deterioration and oxygenation continued to decrease after continuous MV for 10 days before ECMO, an emergency MDT was held and we decided to break the routine ECMO contraindications of more than 7 days MV. Finally, the patient was treated successfully, so we believe that the duration of MV before ECMO could be extended, while ECMO is the only chance to treat the patient.

ECMO parameter setting and adjustment is closely related to each center's experience, and has significant impact on the prognosis of patients. Currently there are no unified standards regarding the safe thresholds of carbon dioxide and arterial oxygen levels. However, arterial oxygen saturations, with a better reflection of oxygen delivery, is of more importance than arterial oxygen tension. Dr. Simon J. Finney has put forward a set of lung protective ventilation parameters which is a Vt of <6 mL/kg predicted body weight to maintain a P-plat < 26 cm H_2_O. In all the parameters, except for P-plat and Vt, general requirements include gas flow between 4 and 5 L/min, arterial oxygen saturation SaO_2_ ≥ 85% to 88% or blood flow should be ≥60% of the theoretical cardiac output, PEEP at 5 to 10 mm Hg, P-plat < 20 to 23 cm H_2_O, respiratory rate of 8 to 10/min FiO_2_ set between 40% and 100% depending on oxygenation capacity of artificial membrane.^[13,14]^

These ECMO parameters are not established for H7N9 ARDS patients, and the respiratory rate is set for patient under sedation. We believe that there are some unique characteristics in H7N9 patient. That is viral infection without effective drugs, long ECMO operation time, and so on. So our parameter settings will be adjusted as follows: respiratory rate 21, FiO_2_(%) 60, PEEP 12 cm H_2_O, and auxiliary flow 4.21 to 4.27 L/min. We believe that a little higher PEEP level (>10 cm H_2_O) can prevent further lung collapse after the institution of ECMO. Furthermore, we hold opinion that “awake ECMO” for patients with ARDS may avoid some of the deleterious effects of MV, sedation, and immobilization in the bed. Thus we set a relatively high respiratory rate.

Due to the requirement for vascular access, anticoagulation, and blood-surface interfacing, complications of ECMO are divided into 3 aspects, they are device related, patient related, or anticoagulation related. Bleeding complications are predominant in ECMO. Their incidence is close to 50%.^[17]^ Oxygenator thrombosis was found in the 10th day of ECMO support in our patient, then we promptly replace the oxygenator, and no other ECMO-related complications were found later. According to our experience, we used to systematically change the circuit once a week to avoid significant device complications. And the ECMO circuit should be monitored several times daily by the medical and nursing team caring for the patient and at least once every 24 h by a perfusionist or other ECMO specialist. If any complication occur, a multidisciplinary consultation should be conducted to establish the best therapeutic approach.

A diversity of anticoagulant values could be monitored in ECMO, some focus only on APTT, some on activated cloting time (ACT), anti-Xa activity, or a combination of ACT and APTT, while some others on thrombelastogram only, each protocol has its own advantages and disadvantages.^[13–16]^ There is no international standardized protocol that has been elucidated for the control of anticoagulation for patients on ECMO. We believe that the anticoagulation management in ECMO should be individualized in accordance to patient's condition. Taking this ECMO patient as an example, though it is the first case that had undergone mechanical heart valve replacement, during ECMO we use heparin as the only anticoagulation, and ACT was maintained at 150 to 180 s during ECMO. Thus we can make both his mechanical valve and the ECMO devices work well. And we adjusted his anticoagulation status based on the occurrence of clotting complications.

V-V ECMO weaning is generally divided into 2 steps. The first one is weaning trial, in which weaning of v-v ECMO should be considered when pulmonary function has improved to 21% FiO_2_ and gas flow <1 L/min on ECMO for several hours. The second step for V-V ECMO weaning should be considered when PaO_2_/FiO_2_ ratio >200 mm Hg, Vt 6 mL/kg, P-plat < 25 mm Hg, FiO_2_ <60%, PEEP <10 mm Hg, pH > 7.3, and echocardiography reveals no evidence of severe acute cor pulmonale. ECMO was weaned from our patient when he had a significant improvement in clinical (notably in his respiratory compliance), blood gas, radiological and ultrasonic aspects on the 16th day after his admission.

H7N9-associated ARDS carries a high morbidity and mortality rate and ECMO support can help treating this life threatening disease. Even if some patients finally survive, returning to work is often limited or delayed,^[18]^ and there can be significant psychological sequelae.^[19]^ Although our patient had no serious complications during treatment, his life quality is not high after that, and he is still unable to return to work. Follow-up of chest CT 1-year later showed serious lung damage, which is difficult to be reversed (Fig. 1D).

## 4 Conclusions

This patient is 1 of the 40 cases of H7N9-caused ARDS in our hospital, because of his special medical history and long duration of MV before ECMO, we report it as a separate case, hoping to provide some reference for ECMO treatment.

## References

[1] Chen Y Liang W Yang S Human infections with the emerging avian influenza A H7N9 virus from wet market poultry: clinical analysis and characterisation of viral genome. *Lancet* 2013; 381:1916–1925. 23623390 10.1016/S0140-6736(13)60903-4 PMC7134567

[2] To KK Chan JF Chen H The emergence of influenza A H7N9 in human beings 16 years after influenza A H5N1: a tale of two cities. *Lancet Infect Dis* 2013; 13:809–821. 23969217 10.1016/S1473-3099(13)70167-1 PMC7158959

[3] Yu L Wang Z Chen Y Clinical, virological, and histopathological manifestations of fatal human infections by avian influenza A(H7N9) virus. *Clin Infect Dis* 2013; 57:1449–1457. 23943822 10.1093/cid/cit541

[4] Combes A Pellegrino V Extracorporeal membrane oxygenation for 2009 influenza A (H1N1)-associated acute respiratory distress syndrome. *Semin Respir Crit Care Med* 2011; 32:188–194. 21506055 10.1055/s-0031-1275531

[5] Australia and New Zealand Extracorporeal Membrane Oxygenation (ANZ ECMO) Influenza Investigators, Davies A Jones D Extracorporeal membrane oxygenation for 2009 influenza A(H1N1) acute respiratory distress syndrome. Jama. 2009;302:1888–1895. 19822628 10.1001/jama.2009.1535

[6] Ius F Sommer W Tudorache I Veno-veno-arterial extracorporeal membrane oxygenation for respiratory failure with severe haemodynamic impairment: technique and early outcomes. *Interact Cardiovasc Thorac Surg* 2015; 20:761–767. 25736272 10.1093/icvts/ivv035

[7] Dellgren G Riise GC Sward K Extracorporeal membrane oxygenation as a bridge to lung transplantation: a long-term study. *Eur J Cardiothorac Surg* 2015; 47:95–100. 24659316 10.1093/ejcts/ezu112

[8] Flecher E Anselmi A Corbineau H Current aspects of extracorporeal membrane oxygenation in a tertiary referral centre: determinants of survival at follow-up. *Eur J Cardiothorac Surg* 2014; 46:665–671. 24574452 10.1093/ejcts/ezu029

[9] Toyoda Y Bhama JK Shigemura N Efficacy of extracorporeal membrane oxygenation as a bridge to lung transplantation. *J Thorac Cardiovasc Surg* 2013; 145:1065–1070. 23332185 10.1016/j.jtcvs.2012.12.067

[10] Wigfield CH Lindsey JD Steffens TG Early institution of extracorporeal membrane oxygenation for primary graft dysfunction after lung transplantation improves outcome. *J Heart Lung Transpl* 2007; 26:331–338. 10.1016/j.healun.2006.12.010 17403473

[11] Gattinoni L Carlesso E Langer T Clinical review: extracorporeal membrane oxygenation. *Crit Care* 2011; 15:243. 22188792 10.1186/cc10490 PMC3388693

[12] Peek GJ Mugford M Tiruvoipati R Efficacy and economic assessment of conventional ventilatory support versus extracorporeal membrane oxygenation for severe adult respiratory failure (CESAR): a multicentre randomised controlled trial. *Lancet* 2009; 374:1351–1363. 19762075 10.1016/S0140-6736(09)61069-2

[13] Richard C Argaud L Blet A Extracorporeal life support for patients with acute respiratory distress syndrome: report of a Consensus Conference. *Ann Intensive Care* 2014; 4:15. 24936342 10.1186/2110-5820-4-15 PMC4046033

[14] Beurtheret S Mastroianni C Pozzi M Extracorporeal membrane oxygenation for 2009 influenza A (H1N1) acute respiratory distress syndrome: single-centre experience with 1-year follow-up. *Eur J Cardiothorac Surg* 2012; 41:691–695. 22228837 10.1093/ejcts/ezr082

[15] Combes A Bacchetta M Brodie D Extracorporeal membrane oxygenation for respiratory failure in adults. *Curr Opin Crit Care* 2012; 18:99–104. 22186218 10.1097/MCC.0b013e32834ef412

[16] Finney SJ Extracorporeal support for patients with acute respiratory distress syndrome. *Eur Respir Rev* 2014; 23:379–389. 25176974 10.1183/09059180.00005514 PMC9487315

[17] Paden ML Conrad SA Rycus PT Extracorporeal Life Support Organization Registry Report 2012. *ASAIO J* 2013; 59:202–210. 23644605 10.1097/MAT.0b013e3182904a52

[18] Hodgson CL Hayes K Everard T Long-term quality of life in patients with acute respiratory distress syndrome requiring extracorporeal membrane oxygenation for refractory hypoxaemia. *Crit Care* 2012; 16:R202. 23082772 10.1186/cc11811 PMC3682304

[19] Risnes I Heldal A Wagner K Psychiatric outcome after severe cardio-respiratory failure treated with extracorporeal membrane oxygenation: a case-series. *Psychosomatics* 2013; 54:418–427. 23756125 10.1016/j.psym.2013.02.008

