# Extracorporeal membrane oxygenation treatment of a H7N9-caused respiratory failure patient with mechanical valves replacement history: a case report: Erratum

**DOI:** 10.1097/MD.0000000000006851

**Published:** 2017-05-05

In the article “Extracorporeal membrane oxygenation treatment of a H7N9-caused respiratory failure patient with mechanical valves replacement history: A case report”,^[1]^ which appeared in Volume 95, Issue 40, the corresponding author did not appear correctly. The corresponding author should have appeared as “Lanjuan Li, Collaborative Innovation Center for Diagnosis and Treatment of Infectious Diseases, the First Affiliated Hospital, Zhejiang University, Hangzhou, China. email: li_doctor@yeah.net.”
